# An evaluation of continuous subcutaneous infusions across seven NHS acute hospitals: is there potential for 48-hour infusions?

**DOI:** 10.1186/s12904-020-00611-3

**Published:** 2020-07-07

**Authors:** J. Baker, A. Dickman, S. Mason, M. Bickerstaff, R. Jackson, A. McArdle, I. Lawrence, F. Stephenson, N. Paton, J. Kirk, B. Waters, J. Ellershaw

**Affiliations:** 1 Pharmacy Department, Royal Liverpool University Hospital, Prescot Street, Liverpool, L7 8XP UK; 2 Palliative Care Institute Liverpool, University of Liverpool, Liverpool, UK; 3 Liverpool Clinical Trials Unit, University of Liverpool, Liverpool, UK; 4 Pharmacy Department, Wirral University Teaching Hospital NHS Foundation Trust, Wirral, UK; 5 Pharmacy Department, Sheffield Teaching Hospitals NHS Foundation Trust, Sheffield, UK; 6 Pharmacy Department, The Christie NHS Foundation Trust, Manchester, UK; 7 Pharmacy Department, St Helens and Knowsley Teaching Hospitals NHS Trust, Prescot, UK; 8 Pharmacy Department, Blackpool Teaching Hospitals NHS Foundation Trust, Blackpool, UK

**Keywords:** Palliative therapy, Subcutaneous infusions, CSCI

## Abstract

**Background:**

Continuous subcutaneous infusions (CSCIs) are commonly used in the United Kingdom as a way of administering medication to patients requiring symptom control when the oral route is compromised. These infusions are typically administered over 24 h due to currently available safety data. The ability to deliver prescribed medication by CSCI over 48 h may have numerous benefits in both patient care and health service resource utilisation. This service evaluation aims to identify the frequency at which CSCI prescriptions are altered at NHS Acute Hospitals.

**Methods:**

Pharmacists or members of palliative care teams at seven acute NHS hospitals recorded anonymised prescription data relating to the drug combination(s), doses, diluent and compatibility of CSCIs containing two or more drugs on a daily basis for a minimum of 2 days, to a maximum of 7 days.

**Results:**

A total of 1301 prescriptions from 288 patients were recorded across the seven sites, yielding 584 discrete drug combinations. Of the 584 combinations, 91% (n = 533) included an opioid. The 10 most-common CSCI drug combinations represented 37% of the combinations recorded. Median duration of an unchanged CSCI prescription across all sites was 2 days.

**Conclusion:**

Data suggests medication delivered by CSCI over 48 h may be a viable option. Before a clinical feasibility study can be undertaken, a pharmacoeconomic assessment and robust chemical and microbiological stability data will be required, as will the assessment of the perceptions from clinical staff, patients and their families on the acceptability of such a change in practice.

## Background

With a recent investigation discovering that one-third of all patients in UK District General Hospitals are expected to be in the last year of life [1], and the projected rise in deaths per year from 468,875 (2014) to 561,000 (2035/36) [2] the challenge of providing adequate end-of life care is daunting. Additionally, with the majority of patients expressing a preference to die at home [3, 4], NHS resources will be placed under increasing pressure to meet the needs of chronically ill patients who live, and want to die, in their usual place of residence [5, 6].

NHS England has predicted the need to find £22 billion worth of savings to balance its books by 2020 [7]. As a result, new ways of providing and structuring services are required to optimise care for patients and make best use of available resources [8]. These pressures, combined with the large number of unfilled medical and nursing posts [9], coupled with the falling number of qualified district nurses (who provide care to patients in their own homes) [10] mean that changes will be required to ensure treatment continues to be provided to the best standard possible.

Continuous subcutaneous infusions (CSCIs) represent an effective method of multiple drug administration in end of life care when the oral route is compromised [11, 12]. Available chemical and microbiological stability data limit the infusion time of a CSCI to a maximum of 24 h [13]. A pilot study conducted at a large UK teaching hospital revealed that in 72% (*n* = 38) of cases, no medication changes were made to the CSCI within the first 48 h of use [14]. This suggests that in certain circumstances (such as patients with stable palliative care needs), there may be an opportunity to deliver medication over 48 h. Such practice may have numerous benefits in both patient care (improving patient autonomy/freedom, enabling clinicians more time to focus on providing compassionate care) and health service resource utilisation.

An investigation was undertaken to gather data regarding the frequency at which CSCI prescriptions are altered in Acute NHS Trusts to identify if there may be a cohort of patients who could theoretically receive a 48-h CSCI. Secondary outcomes included identifying the most frequently prescribed drugs and drug combinations, as well as the assessment of doses prescribed.

## Methods

An open invitation to participate in this evaluation was issued via the UK Palliative Care Pharmacist Network; there were 11 expressions of interest received. Hospital pharmacists or members of palliative care teams at each participating site identified patients who were prescribed CSCIs comprising two or more drugs between July and December 2016.

A nominated pharmacist from each hospital entered the data collected into an electronic database maintained by the Liverpool Cancer Trials Unit. The database was designed to ensure that no patient information was entered. Data relating to a minimum of 50 patients per site was targeted; patients who died or were discharged before 48 h excluded from the study.

### Data analysis

For each patient identified, the healthcare practitioner recorded the drug combination(s), doses, diluent and compatibility for every CSCI daily, for a minimum of 2 days to a maximum of 7 days. Data collection for each patient ceased after 7 days’ continuous treatment, or if the CSCI was discontinued within the 7-day data collection window.

Of primary interest was the number of days that the CSCI ran unchanged. Descriptive analyses were performed, with categorical data presented as counts and continuous data are reported as medians and inter-quartile ranges. The length of an unchanged CSCI prescription in days was compared, across treatment sites and the most prevalent drug types, using regression modelling techniques which account for different levels of variability between patients and between different treatment sites.

## Results

Of the 11 NHS hospitals who expressed interest, one declined participation, two did not reply to the invitation and one failed to obtain Research and Development approval. All sites were in the North of England (Three hospitals within Merseyside, one hospital each in Lancashire and Greater Manchester with the remainder located in South Yorkshire). At the time of data lock, details from 1301 CSCI prescriptions had been recorded (1362 entries were recorded, but 61 excluded due to erroneous entry), yielding a total of 584 discrete drug combinations. Water for Injections was used as a diluent in 766 cases (58.9%), sodium chloride 0.9% as a diluent in 528 cases (40.6%) and no diluent recorded in 7 cases.

### Frequency of prescription changes

An evaluation of the number of days each combination lasted is included in Table 1 for each site. Two hundred eighty-eight patients and 582 combinations are included (2 combinations were removed from this analysis due to incomplete records). 45% (262/582) were changed within 48 h. 38% (221/582) remained stable over 48 h but were changed before 72 h. and 17% (99/582) were unchanged for longer than 3 days. Over a 5-day week (Monday – Friday), 36.7% (214/582) prescriptions remained unchanged for 48 h or longer. This increased to 55% (320/582) when looking at a seven-day week incorporating a weekend. Overall, median duration of an unchanged CSCI prescription was 2 days. The median duration of an unchanged CSCI prescription at sites 3,5, and 7 was, however, less than 2 days.

**Table 1:** Overview of CSCI prescribing across all sites

Site	No. of Patients	No. of CSCI Combinations recorded	No. of CSCIs ran unchanged for one day	No. of CSCIs that ran unchanged for 2–3 days	No. of CSCIs that ran unchanged for 3+ days	No. of CSCIs that ran for 2 or more consecutive days that did not include Saturday and Sunday	Median duration of an unchanged CSCI prescription
1	83	151	56	57	38	41	2 (1, 4)
2	64	128	48	55	25	28	2 (1, 3)
3	11	20	10	8	2	2	1.5 (1, 2.75)
4	26	53	20	25	8	8	2 (1, 3)
5	34	71	39	26	6	9	1 (1, 2)
6	21	35	12	13	10	9	2 (1, 4)
7	49	124	77	37	10	9	1 (1, 2)

Regression modelling was performed to investigate if there were any differences in the number of days a combination was administered which can be attributed to either the site a patient is treated at or any of the drugs which are administered. Table 2 gives the model estimates for the effects of treatment site. Considering the different treatment sties, Sites 3, 5 and 7 all had significantly negative estimates suggesting that combinations are generally administered for shorter periods of time at these sites compared to site 1. Site 4 was also close to statistical significance at the 5% level. Results suggest that the length of time a CSCI were administered were unaffected by the type of drug that was prescribed.

**Table 2:** Results of the mixed log-logistic model to evaluate factors associated with administration time

	Estimate	Standard Error	z value	Pr(>|z|)
Intercept	1.012	0.098	10.290	0.000
Site 1	–	–	–	–
Site 2	−0.118	0.090	−1.316	0.188
**Site 3**	**−0.372**	**0.173**	**−2.152**	**0.031**
Site 4	−0.235	0.123	−1.907	0.057
**Site 5**	**−0.407**	**0.113**	**−3.607**	**0.000**
Site 6	0.003	0.135	0.022	0.982
**Site 7**	**−0.437**	**0.096**	**−4.549**	**0.000**

### Drugs used in CSCIs

Table 3 gives the frequency of each drug along with the mean, median, range and IQR of dose prescribed. The results show that the four most common drugs (midazolam hydrochloride, oxycodone hydrochloride, levomepromazine hydrochloride, morphine sulphate) account for 983/1605 (61%) of all drugs prescribed.

**Table 3:** Frequency and dose range of drugs prescribed in CSCI combinations recorded

Drug	UK Licensing status for SC Infusion	Frequency	Mean dose (mg)	Median dose (mg)	Dose range (mg)	IQR (mg)
Midazolam hydrochloride	Unlicensed	309	11.63	10	(2.5, 60)	(5, 12.5)
Oxycodone hydrochloride	Licensed	230	21.09	15	(2.5, 150)	(7.5, 25)
Levomepromazine hydrochloride	Licensed	225	13.77	6.25	(6.2, 150)	(6.2, 12.5)
Morphine sulphate	Unlicensed	219	19.14	10	(2.5, 190)	(7.5, 20)
Glycopyrronium bromide	Unlicensed	120	1.35	1.2	(0.4, 2.4)	(0.6, 2)
Hyoscine butylbromide	Unlicensed	105	89.9	60	(20, 240)	(60, 120)
Alfentanil hydrochloride	Unlicensed	82	1.8	1.12	(0.2, 12.5)	(0.6, 2)
Haloperidol lactate	Unlicensed	66	2.7	2.5	(0.5, 10)	(1.5, 3)
Cyclizine lactate	Unlicensed	62	150	150	(150, 150)	(150, 150)
Clonazepam	Unlicensed	57	1.18	1	(0.1, 6)	(0.2, 2)
Metoclopramide hydrochloride	Unlicensed	46	36.52	30	(15, 90)	(30, 40)
Ondansetron hydrochloride	Unlicensed	33	15.52	12	(8, 36)	(12, 16)
Ketamine hydrochloride	Unlicensed	13	171.15	150	(100, 300)	(125, 200)
Octreotide acetate	Unlicensed	11	0.65	0.6	(0.6, 0.9)	(0.6, 0.6)
Hyoscine hydrobromide	Unlicensed	11	1.47	1.2	(1.2, 2.4)	(1.2, 1.5)
Dexamethasone sodium phosphate	Unlicensed	10	0.98	1	(1, 1)	(1, 1)
Levetiracetam	Unlicensed	2	375	375	(250, 500)	(312.5, 437.5)
Furosemide	Unlicensed	2	200	200	(200, 200)	(200, 200)
Diamorphine hydrochloride	Licensed	2	7.5	7.5	(5, 10)	(6.2, 8.8)

### Drug combinations used

Of the 584 drug combinations recorded across the seven sites, 128 unique drug combinations were identified. The number of drugs in a combination were distributed as: 2 drugs 251/584 (43%), 3 drugs 232/584 (40%), 4 drugs 98 (17%) and 5 drugs 3 (< 1%). A list of the top 31 drug combinations prescribed is included in Table 4.

**Table 4:** Top 31 combinations present in 1301 recorded CSCI prescriptions

Drug 1	Drug 2	Drug 3	Drug 4	Frequency
Morphine sulphate	Midazolam hydrochloride			40^a^
Oxycodone hydrochloride	Midazolam hydrochloride			35
Morphine sulphate	Glycopyrronium bromide	Levomepromazine hydrochloride	Midazolam hydrochloride	25
Morphine sulphate	Levomepromazine hydrochloride	Midazolam hydrochloride		22
Oxycodone hydrochloride	Hyoscine butylbromide	Midazolam hydrochloride		21
Morphine sulphate	Glycopyrronium bromide	Midazolam hydrochloride		19
Oxycodone hydrochloride	Levomepromazine hydrochloride			14
Alfentanil hydrochloride	Midazolam hydrochloride			13^a^
Oxycodone hydrochloride	Glycopyrronium bromide	Levomepromazine hydrochloride	Midazolam hydrochloride	13
Morphine sulphate	Levomepromazine hydrochloride			13^a^
Oxycodone hydrochloride	Levomepromazine hydrochloride	Midazolam hydrochloride		13
Oxycodone hydrochloride	Metoclopramide hydrochloride			10
Oxycodone hydrochloride	Clonazepam	Haloperidol lactate		10
Oxycodone hydrochloride	Clonazepam			10
Oxycodone hydrochloride	Cyclizine lactate			10
Alfentanil hydrochloride	Glycopyrronium bromide	Levomepromazine hydrochloride	Midazolam hydrochloride	10
Morphine sulphate	Cyclizine lactate			9
Oxycodone hydrochloride	Hyoscine butylbromide	Haloperidol lactate		8
Morphine sulphate	Hyoscine butylbromide	Midazolam hydrochloride		8
Morphine sulphate	Metoclopramide hydrochloride			7^a^
Oxycodone hydrochloride	Haloperidol lactate	Midazolam hydrochloride		7
Alfentanil hydrochloride	Levomepromazine hydrochloride	Midazolam hydrochloride		7
Hyoscine butylbromide	Levomepromazine hydrochloride			7
Oxycodone hydrochloride	Glycopyrronium bromide	Midazolam hydrochloride		7
Glycopyrronium bromide	Midazolam hydrochloride			7
Oxycodone hydrochloride	Cyclizine lactate	Haloperidol lactate		6
Alfentanil hydrochloride	Glycopyrronium bromide	Midazolam hydrochloride		6
Alfentanil hydrochloride	Hyoscine butylbromide	Levomepromazine hydrochloride	Midazolam hydrochloride	5
Morphine sulphate	Hyoscine butylbromide	Levomepromazine hydrochloride	Midazolam hydrochloride	5
Oxycodone hydrochloride	Hyoscine butylbromide	Levomepromazine hydrochloride	Midazolam hydrochloride	5
Oxycodone hydrochloride	Hyoscine butylbromide	Levomepromazine hydrochloride		5

^a^indicates combinations known to have been analysed for 48-h compatibility and stability at clinically relevant doses

An opioid was included in 91% (*n* = 533) of cases with 5 of the 6 most common combinations comprising morphine sulphate. The six most common combinations all comprised midazolam hydrochloride. Twenty-four hour laboratory-tested chemical compatibility and stability data at clinically relevant doses were available for 24 of the top-31 drug combinations [13, 15–25], while 48 h compatibility and stability data were available for 4 of the top-31 drug combinations [16, 22–25].

### Mean morphine equivalent dose

Table 5 gives the mean daily dose of all opioids prescribed and converts to the equivalent morphine dose as per current national guidance [27].

**Table 5:** Mean morphine equivalent daily dose for prescribed opioids

Opioid	Mean parenteral daily dose (mg)	Mean oral morphine equivalent dose (mg)
Alfentanil hydrochloride	1.8	54
Diamorphine hydrochloride	7.5	22.5
Morphine sulphate	19.14	38.28
Oxycodone hydrochloride	21.09	84.36

*N.B Current evidence suggests that doses of parenteral morphine and parenteral oxycodone are equivalent (*i.e. *1:1)* [26]

## Discussion

To our knowledge, this investigation represents the first analysis of CSCI prescribing trends over a continuous period of up to 7 days in the United Kingdom. CSCIs were unchanged following two or more consecutive days in 55% (*n* = 320) of cases, compared to 72% (*n* = 38) in the pilot study [14]. Of the 320 CSCIs in which drugs or dosages did not change for periods greater than 2 days, 33% included the days Saturday and Sunday, which are traditionally less well staffed in NHS establishments. Despite this, there is a potential population in whom a 48-h CSCI infusion may be practicable both in terms of decreasing the frequency of interventions administered to the patient and optimising the utilisation of a healthcare professional’s time.

As demonstrated by the most recent survey of national CSCI prescribing practice, oxycodone was the most commonly prescribed opioid identified by this study [28]. This is despite current national guidance recommending the use of an opioid with the lowest acquisition cost first-line (currently morphine sulphate) [29]. However, morphine sulphate is still widely utilised as shown by its inclusion in five of the six most frequently prescribed drug combinations.

The top two most-common drug combinations correspond with the findings of the most-recent survey of national prescribing practice. The third most-common drug combination included the “four core” drugs needed for quality care of the dying patient (morphine, midazolam, levomepromazine and glycopyrronium) [30]. The frequency of this prescription, and that its use was restricted to one site (site 7) is anomalous and reasons for this need investigation in future work. From the clinical experience of the authors, such patterns can occur with clinicians that have limited experience of prescribing CSCI’s. This was also evidenced by frequent, small, incremental dose changes being made to a patient’s CSCI prescription. The fourth most-common combination (morphine + midazolam + levomepromazine) as found by this investigation was sixth most common in the previously completed evaluation.

Of the top-31 drug combinations, only 4 combinations have 48-h chemical compatibility and stability data. However, as 48-h infusions may present a greater patient infection risk than 24-h infusions, risk/benefit analysis of ward based CSCI compounding versus pharmacy based aseptic preparation, or the inclusion of an in-line antimicrobial filter, [31] will be required prior to adoption into clinical practice.

While there is potentially an alternative to CSCI prescribing (i.e. utilizing agents that possess long plasma half-lives (e.g. levomepromazine) or alternative routes of administration (e.g. transdermal)) these approaches may not be considered appropriate for several reasons. Firstly, the administration of large subcutaneous doses of an agent may result in increased incidents of adverse effects and injection site irritation. Additionally, the duration of action of most if not all drugs commonly administered by CSCI in palliative care does not exceed 24 h. Therefore, a daily visit will still be required.

Secondly, should a dose need adjusting because, for example, the patient’s condition changed, with a CSCI the infusion can be stopped, and doses changed accordingly. In the case of administration of a high dose of a long-acting drug, or a transdermal preparation, the effect will persist for a significantly longer duration.

For these reasons, 48-h CSCIs may be preferred due to the provision of a consistent, drug plasma level.

### Strengths and limitations of this investigation

This evaluation was the first of its kind, and as such has identified opportunities for further research into the utility of extended-duration CSCIs. The adoption of increased infusion duration may have potential to benefit the utilisation of NHS resources and ultimately patient care.

Obtaining local Research and Development approvals at some sites took unexpectedly longer than planned and as a result limited the volume of data collected. We were unable to ascertain from all participating sites whether patients included in the evaluation received a daily review (and whether such a review was performed by a specialist or generalist). Additionally, information as to why the dose remained unchanged following review by a member of the palliative care team each day was not recorded. In clinical practice, however, nursing staff are required to monitor the patient’s symptoms, infusion device and administration set at frequent intervals throughout the 24-h infusion period and request a clinical review if the patient’s condition appears to change significantly. As this evaluation aimed to provide a “snapshot” of prescribing practice at the participating organisations, patient demographics were not collected. Should a national registry be developed, these details would be captured and allow identification of specific patient groups more likely to receive benefit from this intervention.

A further limitation of this investigation was that all acute hospitals that took part were based in the North of England, with a particular focus on the North-West and only performed within an acute hospital setting.

## Conclusions

This evaluation identified patients receiving treatment by a CSCI who did not require changes to drugs and/or dosages for at least 48 h. Thus, there may be a cohort of patients in whom there is the potential to extend the infusion period from the current standard of 24 h to 48 h.

### Future implications for research

To confirm this articles findings and explore populations in which such an intervention may be beneficial, a more robust assessment of practice is required. For example, a future study should also investigate the prescribing of CSCIs in primary care to determine if this follows a similar pattern to that observed in this evaluation. To this end, the creation of a national registry of both primary and secondary care CSCI prescribing may assist in helping to explore the points previously mentioned and identify suitable patient cohorts.

Prior to the undertaking of any clinical feasibility study, pharmacoeconomic analysis and robust chemical and microbiology stability data would be required for all commonly encountered drug combinations. Ideally, such a study would incorporate assessment of the perceptions of clinical staff, patients and their families on the acceptability of such a fundamental change in practice.

## Data Availability

The datasets used and/or analysed during the current study are available from the corresponding author on reasonable request.

## Abbreviations

CSCI: Continuous subcutaneous infusion
IQR: Inter-quartile range
mg: Milligrams
NHS: National Health Service
SC: Subcutaneous
UK: United Kingdom
